# Inkjet printing of TiO_2_/AlOOH heterostructures for the formation of interference color images with high optical visibility

**DOI:** 10.1038/srep37090

**Published:** 2016-11-16

**Authors:** Aleksandr V. Yakovlev, Valentin A. Milichko, Evgeny A. Pidko, Vladimir V. Vinogradov, Alexandr V. Vinogradov

**Affiliations:** 1 ITMO University, International Laboratory “Solution Chemistry of Advanced Materials and Technologies”, Lomonosova 9, 191002, Saint Petersburg, Russia; 2 ITMO University, Department of Nano-Photonics and Metamaterials, Saint Petersburg, Birzhevaya Line d. 14 lit. A, 191002, Russia; 3 Inorganic Materials Chemistry, Department of Chemical Engineering and Chemistry, Eindhoven University of Technology, P.O. Box 513, 5600 MB Eindhoven, The Netherlands

## Abstract

This paper describes a practical approach for the fabrication of highly visible interference color images using sol-gel ink technique and a common desktop inkjet printer. We show the potential of titania-boehmite inks for the production of optical heterostructures on various surfaces, which after drying on air produce optical solid layers with low and high refractive index. The optical properties of the surface heterostructures were adjusted following the principles of antireflection coating resulting in the enhancement of the interference color optical visibility of the prints by as much as 32%. Finally, the presented technique was optimized following the insights into the mechanisms of the drop-surface interactions and the drop-on-surface coalescence to make it suitable for the production of even thickness coatings suitable for printing at a large scale. We propose that the technology described herein is a promising new green and sustainable approach for color printing.

Throughout the history of mankind, different approaches have been developed to extend the limited color sets towards extended color palettes suitable for painting and printing. In modern printing applications, full color images are commonly produced using the standard CMYK and RGB schemes, in which specific colors are generated by combining cyan, magenta, and yellow or red, green and blue base colors, respectively. Conventionally the basic colors for these standard palettes are provided by inks usually containing highly toxic inorganic components including Cd^2+^, Cr^3+^, PbO, Pb_3_O_4_ etc^1^. Not only the disposal of the resulting print products but even their discoloration with time results in the pollution of the environment with these harmful elements and their accumulation in living organisms^2^,^3^. The associated environmental concerns and the challenges with recycling of the common printed goods motivate scientists to search for new green printing technologies and novel approaches for the production of long-lasting color images.

An attractive alternative to the classical chemicals-based ink approaches are the new nanostructuring methodologies based on purely optical phenomena^4^,^5^. The most prominent examples of such phenomena is light interference in thin-layers of surface heterostructures, which is responsible for the coloration of insect wings^6^ or oxidized metal surfaces^7^. Man-made technologies based on similar principles have also been previously developed for the production of color images and coatings. Most of the currently available methods utilize spin-coating or vacuum deposition methods to create interference structures for color control. Recently, we reported a new environmentally benign approach for the production of interference color images by inkjet printing of TiO_2_ nanostructures on various substrates with low refractive indices^8^.

Despite the progress made in the field in the recent years, further developments are needed to reach a practical applicability of the the printing technologies based on the light interference within layered surface structures. In particular, the optical and mechanical properties of the deposited heterostructures need to be optimized in order to address one of the principle challenges of such technologies that is the low visibility of the interference colors. Because of the high refractive index of the applied material and the relatively high reflectance from the interface between its surface and air (Fig. 1), we not only see a color image created by the interference in the thin layer, but also the background reflection from the air-surface interface (Fig. 1, Background and interface reflection). This effectively decreases the intensity of the color produced and, accordingly, limits the optical visibility of the printed image.

In the context of the technology described by us earlier^8^, we hypothesize that an improved color visibility could be achieved through the formation of a layered heterostructure, in which an antireflection layer is introduced on top of a highly refractive base layer. Such a surface structure would allow to establish a balance between the reflectance and interference as is schematically illustrated in Fig. 1. Indeed, the most practical approach to decrease the reflection from the surface is to coat it with a thin layer of a transparent material having a lower refraction index^9^. In the context of inkjet printing, the ideal candidate for such a coating is nanoparticulate alumina or boehmite (AlOOH). Thanks to its relatively low optical density and a moderately high refractive index (RI = 1.655^10^, for pure boehmite), which can readily be reduced by controlling particle size and packing density^11^, boehmite coating allows achieving high difference in refractive indices in the interference structure needed for the high visibility of the interference color prints. Furthermore, this material is expected to be fully compatible with the titania base ink within the perspective inkjet application as suggested by previous studies demonstrating the utility of both materials for the fabrication of optically transparent coatings^12^.

Thus, the envisaged approach towards highly visible interference color printing starts with the generation of an image on a support via the the deposition of a titania layer, which thickness defines the coloration (Fig. 1A). The subsequent coating with a thin boehmite layer decreases the intensity of the “background” reflections and enhances the optical visibility of the color print (Fig. 1B). In this work we for the first time demonstrate the utility of such a concept of antireflection coating to increase the interference visibility by using a desktop inkjet printer and special sol-gel titania and boehmite-based inks. To be able to create and control surface heterostructures by inkjet printing, we synthesized tailor-made inorganic inks based on titania and alumina sols and optimized their rheological properties to ensure their compatibility with the printhead of the inkjet device. The target properties of the inks were defined by the requirements of the practical applicability that is the high stability of these sols and the possibility to obtain final optical coatings upon drying at low temperatures or preferably at room temperature under ambient conditions. One of the focal points of this study was the optimization of the ink composites to establish such an appropriate sol-gel transition on the surface upon drying. Finally, the drop-surface interactions were studied to ensure the drop deposition without border effects that is necessary for the good printability. The promising results presented in this work demonstrate the high potential of the described technology and the underlying physico-chemical principles for the manufacture of foil or polymer materials in a roll-to-roll manner with the inkjet color printing. We argue that this approach holds a promise to pave the way towards a new green and environmentally benign universal printing technology alternative to the current pigment-based printing.

## Results

### Ink properties and advantages of the sol-gel approach

**Figure 2:**
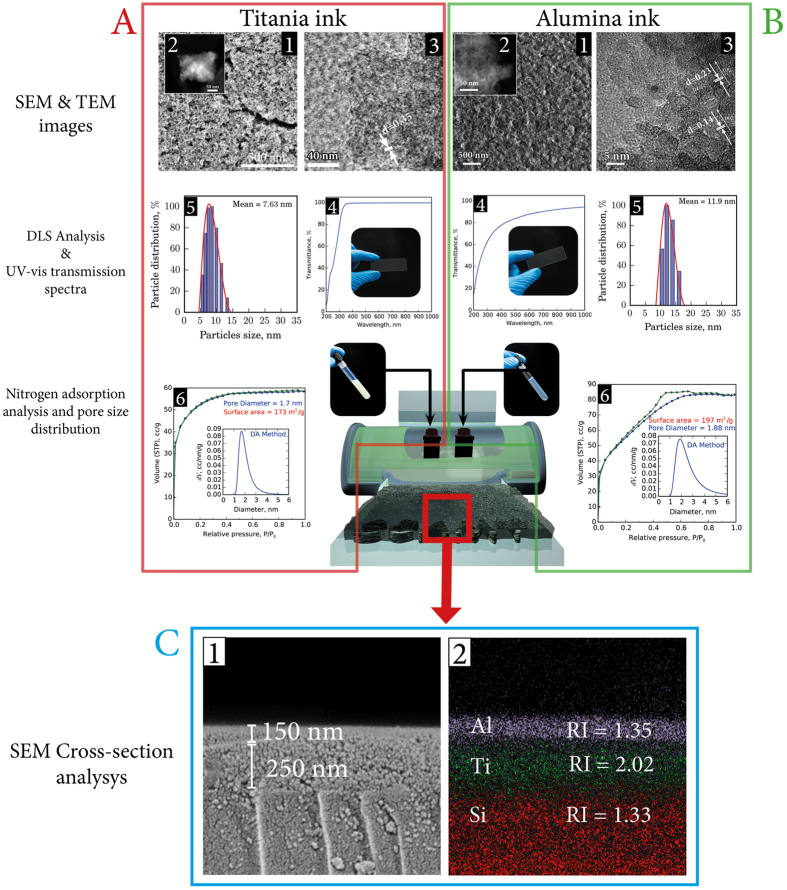
The key characteristics of the inks and the resulting coatings of titania (**A**) and alumina (**B**). 1) SEM images of the surface coatings with 2) STEM of the nanoparticles shown as inserts; 3) HRTEM images of the nanoparticles in the as-synthesized inks; 4) UV-Vis transmission spectra of the thin films on a glass slide together with the respective photograph shown as an insert; 5) the particle size distribution characteristics of the sol ink as revealed by the dynamic light scattering measurements; 6) nitrogen adsorption-desorption isotherms and the respective DA pore size distributions (inserts) of the xerogel formed by the inkjet printing. Panel (C) presents the results of the SEM cross-section analysis of the deposited inorganic layers on a glass substrate (1) and the respective EDX mapping (2) with the refractive indices shown for the alumina and titania layers as well as for the glass substrate.

The availability of the suitable ink materials and their optimal characteristics for inkjet printing of 2D photonic nanostructures are the prerequisites for the development of the target technology. In this work we eployed two ink materials based on the nanocrystalline sols of titania and alumina. Main characteristics of the inks as well as the resulting coatings are summarized in Fig. 2.

To synthesize these inorganic inks, we employed the low-temperature sol-gel process resulting in stable titania and alumina sols. Previous studies demonstrated that such a methodology can be utilized for the preparation of stable nanocrystals with high zeta-potential suitable for storing under ambient conditions for prolonged periods of time^13^. The synthesized sols contained uniform nanoparticles with average sizes of 11.9 ± 0.2 nm for TiO_2_ and 7.6 ± 0.3 nm for AlOOH (Fig. 2A,B, panels 6) having the zeta-potential of +36 mV (Figure S1) and +45 mV, respectively. Both materials were characterized by high optical transparency, crystallinity and the near bulk porosity (Fig. 2A,B, panels 3, 4, 6).

**Figure 3:**
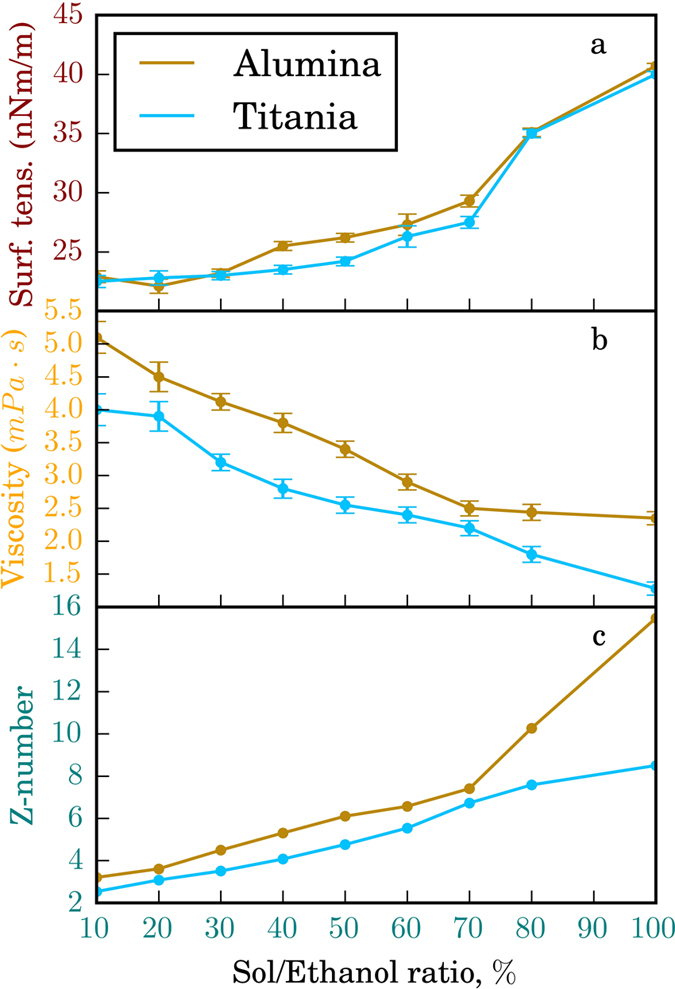
The impact of ethanol dilution on the viscosity (**a**), surface tension (**b**) and Z-numbers of titania and alumina sols. (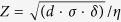), where *δ* is the density, *d* is the diameter of the nozzle, *σ* is the surface tension, and *η* is the viscosity).

The ink material for interference inkjet printing should produce dense surface structures to achieve high optical characteristics. This implies that the solids formed upon drying of the ink droplets must contain no organic residue. This limits dramatically the applicability of the conventional approaches based on the use of organic surfactant and surface tension modulator additives for controlling viscosity and surface tension of inks. An important advantage of the current nanoparticle-based sol materials is that their respective characteristics can be readily adjusted by simply varying the pH of the suspensions in ethanol-water mixtures^14^. Here, we aimed at the application of the inorganic inks in desktop inkjet printing devices, which unlike their more advanced research counterparts do not allow to directly monitor the drop formation and to adjust the voltage waveform. Instead, we employed an alternative indirect approach to guide the optimization of the inks. The key physical parameters of printing fluids are viscosity, density, and surface tension as they influence the mechanism of the drop formation and its size. In inkjet printing, the reciprocal of the Ohnersorge number, also referred to as the Z-number, is used to assess the overall printability of printing fluids^15^. The influence of the solvent medium composition on the surface tension, viscosity and Z-number characteristics of the alumina and titania-based sol inks is shown in Fig. 3.

The Z-number was used as an indicator of droplet formation and printability that account for capillary break-off length and time, droplet volume, and satellite formation. For both inks, the measured Z values were in the range between 4 and 9 that matches perfectly with the values necessary to achieve good range of printable rates for different printheads^15^.

The printability of the inks was further confirmed by forming well-defined coatings. The base titania layer has a uniform structure and a low level of surface cracks Fig. 2A^1^.

The existing cracks on the titania coating were the result of the dense packing of small crystalline nanoparticles (Fig. 2A^3,5^, Figure S3) effectively locking the residual solvent molecules, which create an additional stress within the layer upon drying. The porous structure of the inorganic layer lacking large pores (Fig. 2A^6^) gives rise to a high transparency to visible light (Fig. 2A^4^) and at the same time is highly beneficial for the refractive index of the base coating. On the other hand, the mesoporous structure of alumina is expected to be beneficial for the optical characteristics of the anti-reflective coatings. It allows to effectively reduce the refractive index via low-density packing of porous nanocrystalite rods (Fig. 2B^6^). Because of the high uniformity of the nano-crystallite sizes (Fig. 2B^3,5^, Figure S3) in the inks, the resulting coatings were characterized by an almost complete transparency in the visible range and at the same time by a low refractive index. These optical features will be discussed below in more detail.

The uniform composition and high tunability of the current sol-based inks allow forming dense coatings characterized by structures with a small amount cracks and constant refractive indices. SEM micrographs of the cross-section of the optical heterostructures produced by inkjet printing of the titania and alumina inks are presented in Fig. 2C. Taken together the results in Fig. 2 indicate the possibility of the precise control over the formation of heterostructures made of solid surface composites, which do not contain large pores and internal cavities, while they exhibit clear boundaries and are transparent to visible light that is sufficient to achieve the desired optical effect.

### Sol-gel transition and uniform layer formation

Driven by the need to further optimize the technology, we further focused on the investigation of the processes underlying the sol-gel transitions on the surfaces. The strong adhesion between the printing material and the support is the fundamental phenomenon in the inkjet printing.

**Figure 4:**
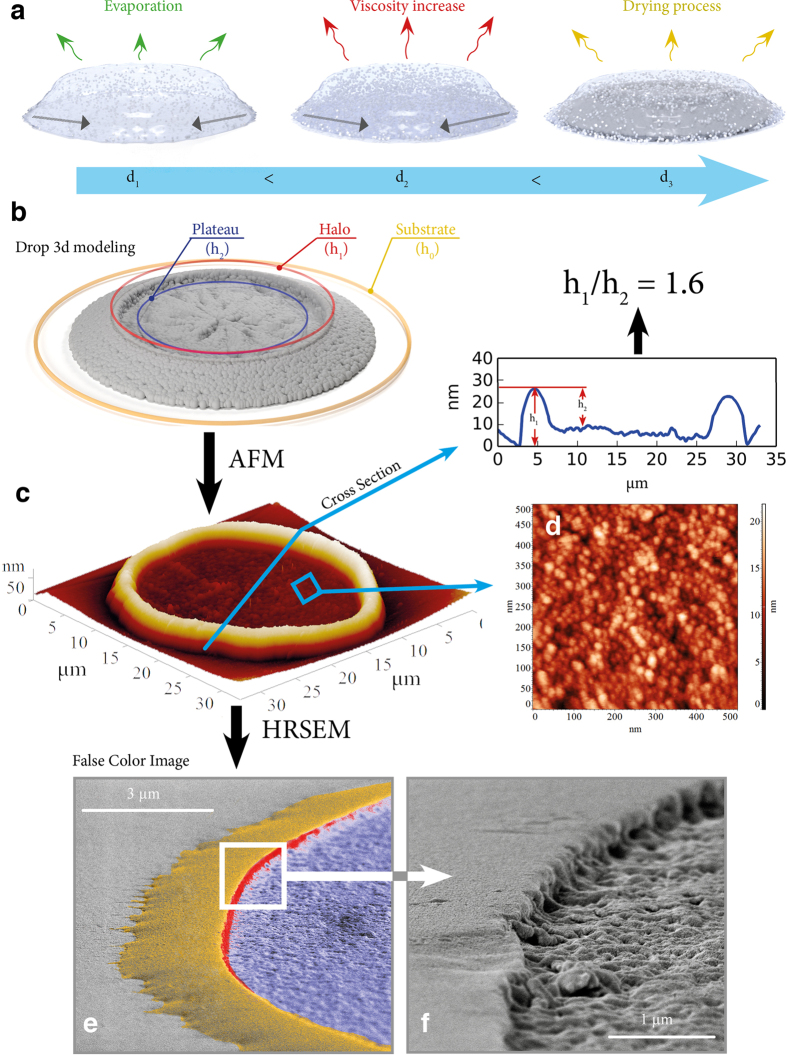
Visualisation of the formation of the solid surface structure and the sol-gel transition from a single drop formed by inkjet printer on the glass surface. (**a**)a model of the drop solidification upon drying accompanied by the formation of a pie-drop structure (**b**), which can be measured by atomic force microscopy (**c**) and scanning electron microscopy to determine the nanoscale structure of the drop. AFM roughness measurements evidence the coffee-ring effect evident from a substantial difference in the height of the edge and the inner parts of the drop with respect to the substrate. False color SEM (**e**) and HRSEM (**f**) images of the drop on the glass substrate further confirm the coffee-ring effect on the drop.

Figure 4 illustrates schematically the key stages of our inkjet printing approach, in which the printed layer formation and the strong adhesion to the support takes place through the sol-gel transition. When the ink droplet reaches the support, the surface-liquid interaction emerges and the solvent evaporation begins. Solvent removal increases the viscosity of the droplet resulting ultimately in the sol-gel transition producing a solid structure on the surface. An understanding of the underlying processes and the complexity of the interactions between the components is needed to achieve a good control over the printing process and to ensure a high optical quality of the final print. Unlike the classical inkjet printing based on the phenomenon of absorption, the refractive color printing involves the interactions of non-adsorbent surfaces.

Of particular importance to the current technology are the coffee-ring effects, which depending on the conditions and the nature of the interacting entities can have a positive effect on the characteristics of the final prints or cause damage to the surface structures^16^,^17^. The coffee-ring effects in the case of nanocrystalline ink printing of optical structures can potentially lead to the formation of raw borders at the droplets resulting in the low overall quality of the optical coatings and hamper the formation of well-defined heterostructures. The coffee-ring effect is almost inevitable when the ink droplets are applied to hydrophobic polymer or glass surfaces. However, we found that the water-alcohol mixtures of peptized sols after sol-gel transition allow creating flat and largely homogeneous surfaces having dense structures and featuring only a small coffee-ring effect.

Figure 4 shows the results of the 3D modeling of a sol drop on a surface together with the AFM and HRSEM characterization of a single droplet formed by inkjet printing on a glass support. After the sol-gel transition a rather uniform surface structure was formed with a diameter of *ca.* 30 *μ*m and an average thickness of *ca.* 10 nm. The border region was represented by a halo approximately twice thicker that the plateau in the middle of the droplet.

**Figure 5:**
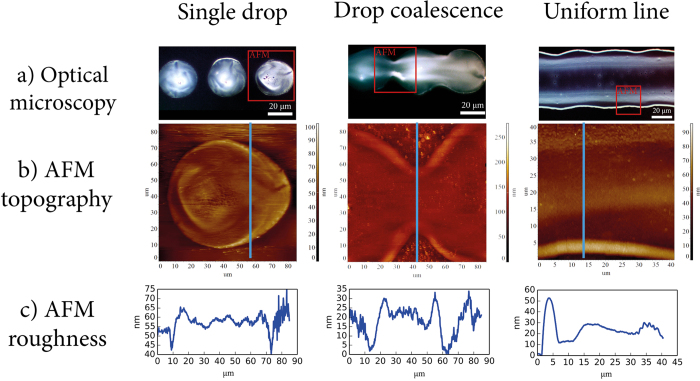
The coalescence of ink droplets allows the formation of a nearly uniform surface on a flat substrate with a thickness deviation of no more than 20–30 nm along the Z axis. In the experiments, droplets of titania sol with varied volumes (2, 5, and 10 pL) were deposited on the glass surface. The drop size was differentiated by changing the grayscale brightness of pixels. Here we use optimal ink solution with 30 vol. % of the stock TiO_2_ sol with surface tension 23.0 ± 0.4 mN/m and viscosity of 3.2 ± 0.2 mPa⋅s. The drop-on-demand inkjet printing (DOD) with room-temperature drying allows to create three stages of drop-on-surface interaction–single drops, coalescence and line formation. The resulting structures were characterized by optical microscopy (**a**) and atomic force microscopy (AFM, **b**). AFM provides information about the cross-section (**c**) of border effects and places of interaction between drops from which the roughness of the surfaces can be assessed.

More uniform surface structures could be manufactured by applying the principle of drop coalescence, which is illustrated in Fig. 5 with an example of the titania layer formation on a flat surface. The application of drops with a sufficient frequency and volume to the surface gives rise to the coalescence between the drops and results initially in the formation of connecting bridges and, with increasing drop volume, ultimately produces a uniform line with no coffee-ring effect along its length. In the course of droplets coalescence, joint structures develop and the coffee-ring is being effectively displaced to the borders of the newly formed extended pre-line structure. These results imply that upon the coalescence of a large number of droplets, the conditions can be met when the edge effects occur predominantly at the macroscopic boundaries so that they do not affect the properties of the operating part of the heterostructure.

**Figure 6:**
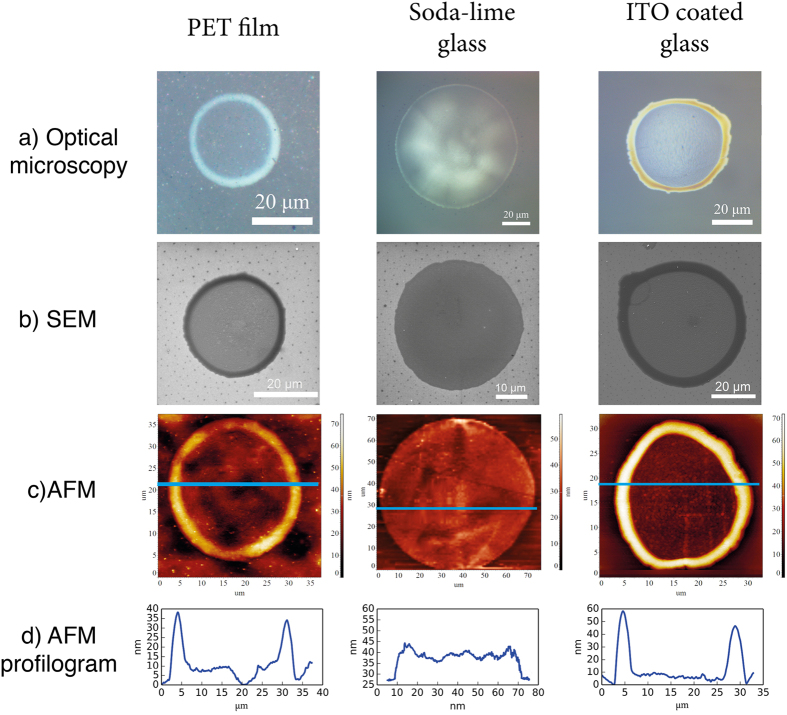
Formation of a solid drop after applying titania droplet by inkjet printing on the surface of substrates with different nature. The figure presents the images obtained by optical microscopy (**a**), scanning electron microscopy (**b**) and measured by atomic force microscopy (**c**) with a respective roughness cross-section plot (**d**).

The geometric parameters of the solid structure formed after the sol-gel transition in ink droplet are determined by the initial properties of the solvent and the actual printing conditions such as the drying temperature. The nature of the support has only a limited influence on the dimensions and the properties of the deposited droplets. Figure 6 shows the inkjet solid drops on different surfaces with varied hydrophobicity. Importantly, even when deposited on supports with a relatively high hydrophobicity such as ITO coating or polymer surface evenly-sized drops could be formed (Fig. 6) which did not break into smaller ill-defined droplets or produce raw xerogel surfaces.

This could be achieved because the viscosity of the deposited liquid increases rapidly upon drying preventing thus from pulling the thin liquid layer apart. This allows creating highly uniform droplets virtually lacking the coffee-ring effects when applied to less hydrophobic surfaces such as glass. Even in the presence of the coffee-ring effect, the edge of the droplet largely retains its optical properties. Nevertheless, the possibility to adjust the surface tension and viscosity of the current sol-based inks by varying the solvent composition (particularly, the alcohol content) together with the available methods to alter the hydrophobic properties of the supports (for example, the formation of plasma-treated hydrophilic surfaces such as surface-sized papers^18^, Si and SiO_2_^19^ and polymers^20^ render these compounds highly attractive as the basis for the universal inks applicable to different substrates.

The fast drying of ethanol-rich titania ink resulting in a thin-film deposits creates a possibility to increase uniformly the thickness by multilayer deposition when using the same cartridge in the subsequent print or for constructing multilayer heterostructures by applying another ink from the next cartridge. In our experiments we demonstrated the utility of the second approach as is shown in Fig. 2 by applying the alumina ink on top of the titania print from the second cartridge. The two phases were clearly separated and no mixing of the layers could be observed, because the base titania layer had dried completely at the time of the alumina deposition and its structure had a clear layer boundary. The hydrophilic nature of the titania surface is suitable for the efficient interaction with the alumina ink drops. This favors the formation of a smooth second layer without a notable coffee-ring effect.

### Optical properties of heterostructure

Having established the principles governing the formation of the structures during the inkjet printing of sol-based inks, we focused on the analysis of the optical properties of the resulting heterostructures. The optical properties of the interference heterostructures are largely determined by the difference in the refractive indices of the substrate, the interference layers and anti-reflective coating. To control and improve the optical properties of the printed patterns, we made use of the anti-reflection coating effect while maintaining the balance between reflection, transmission and the interference.

**Figure 7:**
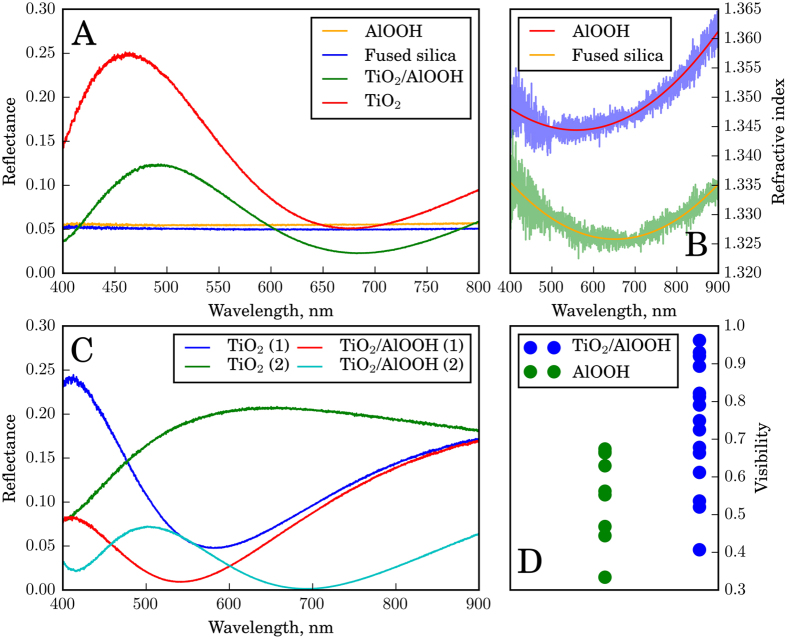
(**A**) Reflectance at normal incidence of the optical radiation onto a TiO_2_ film placed between two dielectric media (2 mm plate of fused silica and air or AlOOH layer) as a function of wavelength. Black and red curves correspond to reflectance at normal incidence of the optical radiation onto 2 mm fused silica and 0.5 mm AlOOH layer; (**B**) Dispersion of refractive indices of fused silica and AlOOH within the visible spectral range obtained in case of one reflection border; (**C**) Reflectance at normal incidence of the optical radiation onto a TiO_2_ film placed between two dielectric media (2 mm plate of fused silica and air or AlOOH layer) as a function of wavelength. Indices (1) and (2) denote different film thickness; (**D**) Visibility V of interference fringes in reflected light for TiO_2_ and TiO_2_/AlOOH films. *V* = (*I*_*max*_ − *I*_*min*_)/(*I*_*max*_ + *I*_*min*_), where *I*_*max*,*min*_ means intensity of reflected light in minima and maxima. Average values of V within the entire spectral range for TiO_2_ and TiO_2_/AlOOH films are 0.56 and 0.74.

Figure 7A illustrates the wavelength-dependence of the reflectance at normal incidence of light onto a TiO_2_ film placed between two dielectric media (2 mm plate of fused silica and air or AlOOH layer). The films appear colored because of the light interference that is due to the finite thickness of the surface layer having a refractive index different from that of the environment. Under ambient conditions on air, at a certain wavelength the minima in the reflection spectra correspond to the destructive interference of light reflected from the air-film and film-silica interfaces. Independent of the thickness of the film the reflection coefficient is determined only by the refractive indices of air and fused silica as is given by:


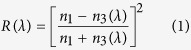


where *n*_1_ = 1 is the refractive index of air and *n*_3_(*λ*) is the refractive index of the fused silica. When the environment is created by the AlOOH layer, we observe a zero reflection Fig. 7C. This can be rationalized by taking into account the similarity between the refractive indices of the thin AlOOH layer and that of the fused silica (Fig. 7B) so that the reflection of light from the resulting layered structure with *n*_1_ = 1.35 and *n*_3_ = 1.33 is accompanied by a substantial decrease of its intensity in agreement with the equation (1).

On the other hand, the reflectance maxima in the spectra shown in Fig. 7A correspond to a positive interference of the reflected light with certain wavelengths. Here, independent of the film thickness, the reflection coefficient at these wavelengths is determined by:


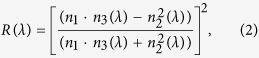


where *n*_2_(*λ*) is the refractive index of TiO_2_ film at the same wavelengths (*n*_2_ ≈ 2). When the environment is provided by the AlOOH layer rather than by air the reflectance decreases substantially (Fig. 7C) again due to the similarity of the refractive indices of the terminating phases, namely the fused silica support and the AlOOH antireflective layer. However, this decrease in reflectance is accompanied by the visibility enhancement of the interference fringes as can be seen from Fig. 7D. This makes the reflected light with positive interference look more contrast. Quantitatively, the introduction of the antireflective AlOOH layer increases the contrast by 32%, which in practice means that the interference picture should become much more “visible” to a naked eye.

### Inkjet printing of interference solid thin films

**Figure 8:**
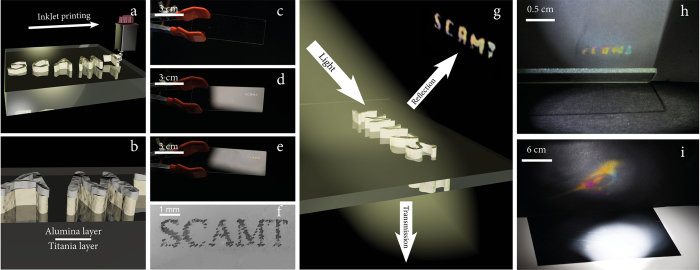
Scheme for the production of a reflective coating; Schematic diagram of a single-pass method (**a**) for deposition a layer of titania as a base layer as was described before. Formation of a double layer heterostructure (**b**) by application of alumina on top of titania layer and creating transparent structure (**c**) but already colored in reflectance from single titania layer (**d**) on (small SCAMT writing) and improved visibility of colors by application of AlOOH layer (**e**) on top (SCAMT writing). Even on microlevel clear structure can be produced by single drops and on the maximum resolution of the desktop printer (**f**) as a perfectly clearly seen on SEM image of droplets deposited on the slide in the form of a writing. Produced structure reflect color image (**g**) on the slide but transparent on transmission, allowing for automatic reading in reflection or projection of microimages in reflection printed writing from the slide (**h**) or in big image reflected from the polymer substrate (A4 format).

Next, we focused on the practical realization of the above fundamental concepts. At this stage, we should emphasize the fundamental differences in the optics behind the color images and graphics formed by surface heterostructures and based on the physical principles of interference. The principle difference with the conventional techniques where the light absorption characteristics define images is that in the interference printing the colors are seen in the reflection mode. As a result, the enhanced interference printing technique should follow a different approach to image rendering, which at the same time may allow generating printed patterns with unique optical properties (Fig. 8).

We have inkjet printed images on the surface of a glass slide or polymer A4 sheet in a single pass (using two cartridges containing TiO_2_ and AlOOH inks). Such an approach allows producing double layered structure (Fig. 8b) with pronounced color reflections (Fig. 8g). The images obtained in this way are fully transparent (Fig. 8c), but can be used to project reflection with enhanced brightness, for example on a wall or on a screen (Fig. 8g–i). We propose that such an imaging approach based on the reflections from the substrate and the fundamental possibility of projecting printed images on large areas aided by focusing optics can have a direct practical importance in the fields of information encoding, copyright protection and automatic reading or visualization.

**Figure 9:**
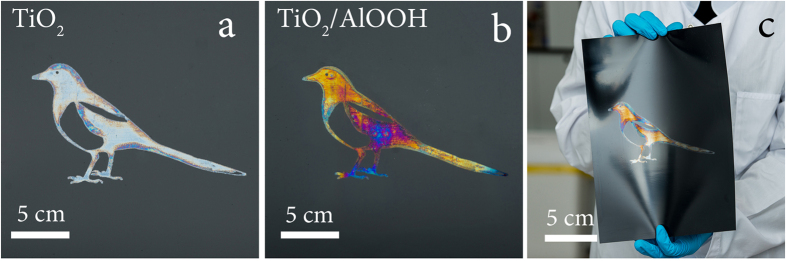
Photographs of interference color images on a polymer substrate: (**a**) image with titania layer; (**b**) image on a polymeric substrate with a two-layer titania/alumina structure; (**c**) curved polymer substrate with a color image.

Compared to the other methods for creating optical nanostructures, inkjet printing is particularly attractive in view of the possibility of the fast production of large-scale optical structures and complex shapes on different substrates. We postulate that this method can be used to obtain full-color refractive images or structures resembling hot stamped metal foil reflectors on various substrates without the need for an additional annealing step. This extends the application range for such systems. A fundamentally new approach for creating uniform photonic structures based on the sol-gel ink transition allows to control the associated optical effects. Such structures can be created on the surface of polymer films resulting in full-color refractive image prints characterized by high flexibility and visibility at any angle at diffuse ambient light (Fig. 9).

## Conclusion

To conclude, in this work we presented the development of a practical approach for the visibility enhancement of color interference imaging by heterostructure formation on solids using sol-based inks and desktop inkjet printer. By studying the sol-gel transition process on the surface we obtained an important insight into the morphology control of the individual surface droplets. By simultaneously optimizing the ink composition and the surface hydrophobicity one can establish optimal conditions for the uniform droplet formation on various supports. The substantial enhancement of the color visibility of the interference prints was achieved via a layer-on-layer deposition method based on transparent materials with different refractive indices, namely, TiO_2_ and AlOOH. The resulting prints are completely transparent, while the images can be visualized by projecting them in a reflection mode. Optical nanostructures produced using this approach have a wide range of applications due to the high thermal stability and with colors protected from fading. The technological process formulated in this work utilizes only non-critical, cheap and environmentally benign materials rendering it a highly attractive green printing approach that may open avenue towards alternative applications requiring the construction of optical devices by fast, green and versatile technology.

## Methods

### Chemicals

The chemicals in this study were used as purchased: titanium (IV) isopropoxide (TTIP, 97%), nitric acid (HNO_3_, 65%), aluminum isopropoxide (>98%, Aldrich). All reagents were purchased from Sigma-Aldrich. 96% ethanol was purchased from ChimMed, Russia. All aqueous solutions were prepared by using highly pure water from Millipore Elix (15 MOm/cm^3^). Soda-lime glass plates were used as substrates (microscope slides 26 mm × 76 mm, Paul Marienfeld, Germany). Polymeric substrate are pre-cut A4 PET film sheet (ChimMed, Russia). Indium tin oxide coated glass slide, rectangular 15–25 Ω/sq, slide (Aldrich). Substrates were sonicated in USI bath, rinsed with isopropanol, and dried in a flow of air.

### Synthesis of the TiO_2_ sol

To produce a sol, two solutions were prepared. For the first solution, 16 mL of titanium isopropoxide were dissolved in 12 mL of 2-propanol forming a clear solution. To prepare the second solution, 0.7 mL of nitric acid was added to 100 mL of deionized water, and the mixture was heated to 70 °C, after which the first solution was slowly added to the second one upon stirring. The resulting mixture was stirred for 1 hour at 80 °C, after which it was sealed with plastic lid and incubated for 1–2 weeks at room temperature and under stirring.

### Synthesis of the TiO_2_ ink

The synthesized sol solution was at first evaporated in a rotary evaporator under reduced pressure at 50 °C to bring pure TiO_2_ to a solid phase concentration of no less than 8 wt. %. To change the surface tension and viscosity, the aqueous solution of the sol was mixed with ethanol, and a series of printing formulation candidates with different rheological properties was prepared and denoted. The resulting solution was homogenized for at least 12 days. Analyzing the data obtained, inkjet-ready colloidal systems with no visible gelation (Figure S1) in the range of appropriate Z-numbers. According to a plot of hydrodynamic radius of the particles and zeta potential vs. ethanol concentration in the original TiO_2_ sol, Figure S3, the ink containing 70% of ethanol was considered an optimal colloid for inkjet printing. It should be noted that the stability of the colloidal solution is greatly reduced by adding more than 70% of ethanol. This is due to the fact that ethanol modifies the structure of the electrical double layer of TiO_2_ particles, sharply decreasing their stability.

### Synthesis of the AlOOH sol

Alumina sol was prepared according to a reported procedure^21^ by addition of 2.2 g of Al(C_3_H_7_O)_3_ to 50 mL of deionized water at 90 °C resulting in the immediate formation of a white precipitate. Prior to ultrasonic treatment the precipitate was kept at 90 °C under vigorous stirring for 15 min to complete the production of boehmite nanoparticles and to complete the evaporation of the isopropanol formed during hydrolysis. The final suspension was ultrasonically treated for 2 h to form a viscous sol. The resulting sol was cooled to room temperature. Final sol had +45 mV zeta potential providing excellent stability.

### Preparation of the AlOOH ink

The synthesized sol solution was dried under reduced pressure at 50 °C to bring pure AlOOH to a solid phase concentration of no less than 5 wt. %. To change the surface tension and viscosity, the aqueous solution of the sol was mixed with ethanol, and a series of printing formulation candidates was prepared and denoted. The resulting solution was homogenized for at least 12 days. The main rheological properties of the ink are shown in Fig. 3. Analyzing the data obtained, inkjet-ready colloidal systems with the highest stability were identified. According to a plot of hydrodynamic radius of the particles and zeta potential vs. ethanol concentration in the original AlOOH sol, Fig. 3, the ink containing 60% of ethanol was considered an optimal colloid for inkjet printing. It should be noted that the stability of the colloidal solution is greatly reduced by adding more than 60% of ethanol. This is due to the fact that ethanol modifies the structure of the electrical double layer of AlOOH particles, sharply decreasing their stability.

### Inkjet printing of heterostuctures

For inkjet printing of color images, we have used an A4 polyethylene terephthalate (PET) film, 105 *μm* thick, and soda-lime glass plate microscope slides 26 × 76 mm, Paul Marienfeld, Germany. Printing was carried out using two cartridges, in which 8 mL of the TiO_2_ ink and 6 mL of the AlOOH ink were preliminarily placed. The printer driver settings implied the maximum printing quality. Printing was later performed on the same selected substrate by a single-pass method. Desktop office printer Canon Pixma IP2870 with standard cartridges PG-745, CL-746 was used in the experiments. The print head built into the cartridge has a droplet size of 2 pL and 1280 nozzles. After washing, the cartridges was filled with TiO_2_ and AlOOH inks without any modification. The design of the printer and the cartridge remained unchanged.

### Optical measurements

To investigate optical properties of TiO_2_ and AlOOH coatings, ink was printed on polished glass surface (microscope slides 26 mm × 76 mm, Paul Marienfeld, Germany) using Canon IP2480 inkjet printer. After applying, the produced sol was dried at 60 °C in the air for 15 minutes to form a thin layer of TiO_2_.

Then, optical reflection measurement of TiO_2_ films at normal incidence was carried out to obtain refractive indices. For this experiment the confocal optical scheme was arranged Figure S2. The incident unpolarized light from a halogen lamp (HL-2000-FHSA) was focused on the film surface through a 50x microscope objective (Mitutoyo M Plan APO, NA 0.55). Reflected light was collected through the same objective and then analyzed by a spectrometer (HORIBA LabRam HR) with a cooled CCD camera (Andor DU 420A-OE) and a 150 g/mm diffraction grating. The obtained spectra were normalized by the known spectrum of the halogen lamp. The reflectance spectra from different points of the film allowed us to estimate the error for refractive indices at various wavelengths.

### Additional characterization

The samples for transmission electron microscopy (TEM) and high resolution transmission electron microscopy (HRTEM) were prepared by dispersing small amounts of samples in ethanol to form a homogeneous suspension. A drop of the suspension was deposited on a carbon-coated copper grid for HRTEM observations (FEI TECNAI G2 F20 operating at 200 kV). DLS measurement was carried out on Photocor Compact-Z. Samples for SEM and AFM measurements was printed on glass and studied on Extra High Resolution Scanning Electron Microsopy MagellanTM 400 L and NT-MDT Next. Transmission optical spectroscopy was made on Agilent Cary 60 with slide adapter. Low resolution SEM microscopy was take on Tescan Vega 3 microscope, and optical microscopy on LOMO BIOLAM with external CCD camera. Photographs of the samples were taken by the camera Nikon D800 with lens AF-S NIKKOR 24–70 mm f/2.8 G ED and AF-S NIKKOR 70–200 mm f/2.8 G ED VR II without using additional flash. The camera color calibration was performed using X-Rite ColorChecker Passport.

## Additional Information

**How to cite this article**: Yakovlev, A. V. *et al.* Inkjet printing of TiO_2_/AlOOH heterostructures for the formation of interference color images with high optical visibility. *Sci. Rep.* **6**, 37090; doi: 10.1038/srep37090 (2016).

**Publisher's note**: Springer Nature remains neutral with regard to jurisdictional claims in published maps and institutional affiliations.

## Supplementary Material

Supplementary Information

## Figures and Tables

**Figure 1:**
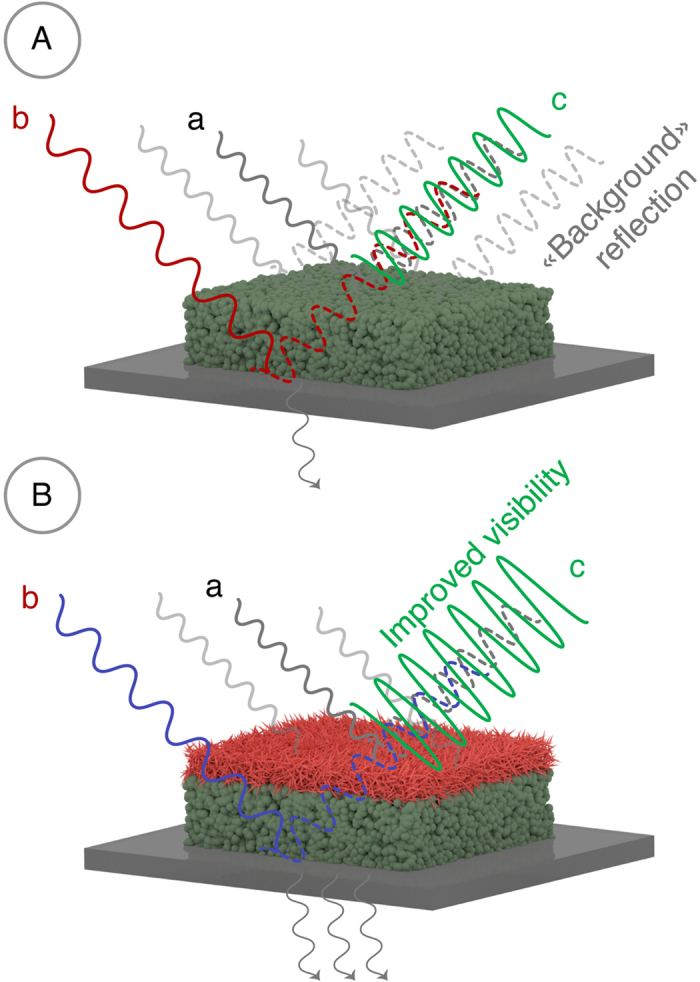
A schematic illustration of the colored interference by a nanoscale titania (Green) thin film (**A**) and the mechanism of the interference visibility enhancement in a surface heterostructure containing an anti-reflective AlOOH (Red) layer on the surface of interfering TiO_2_ film (**B**). The scheme depicts two incident light beams (a and b), which combine upon reflection to produce beam c. The introduction of the anti-reflective layer decreases the “background” reflection and therefore improves the effective visibility of the target beam c.
