# Supplementary material for: Inkjet printing of TiO2/AlOOH heterostructures for the formation of interference color images with high optical visibility
Source: Sci Rep. 2016 Nov 16;6:37090. doi: 10.1038/srep37090 (PMC5111046; doi:10.1038/srep37090)
Supplement: Supplementary Information [file srep37090-s1.pdf]

Supporting information

Inkjet printing of TiO_2/AlOOH heterostructures for the formation of interference color images with high optical visibility

Aleksandr V. Yakovlev,[a] Valentin A. Milichko,[a] Evgeny A. Pidko,[a,b] Vladimir V. Vinogradov, [a] and Alexandr V. Vinogradov[a]

[a]*ITMO University, Saint-Petersburg, Russia*

[b] *Inorganic Materials Chemistry, Eindhoven University of Technology, The Netherlands*

1. Titania particle size and Zeta-potential

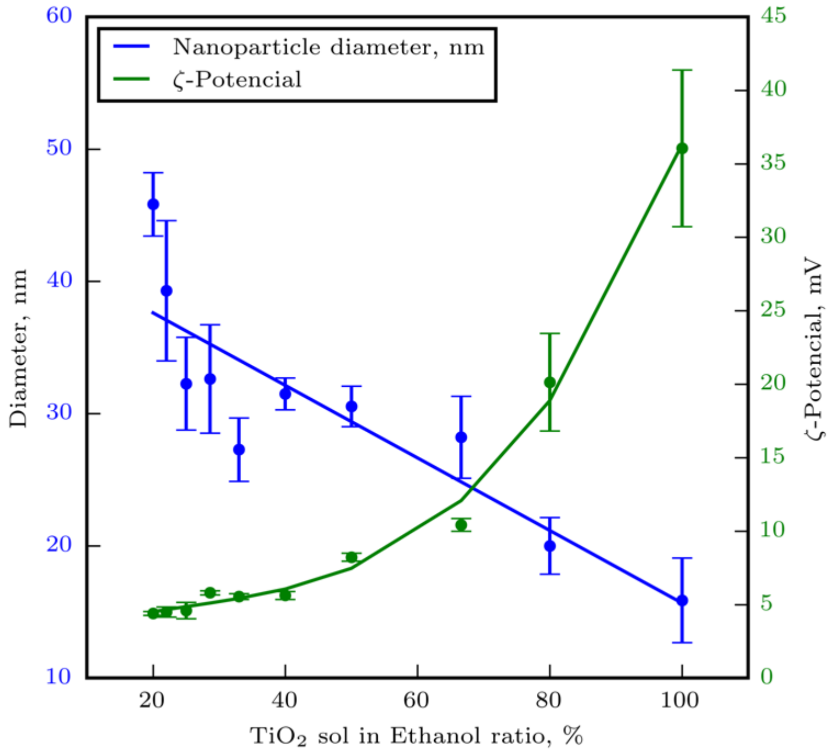

Fig. S1. Nanoparticle diameter and Zeta-potential in depending on the sol/ethanol ratio.

2. Optical measurement setup

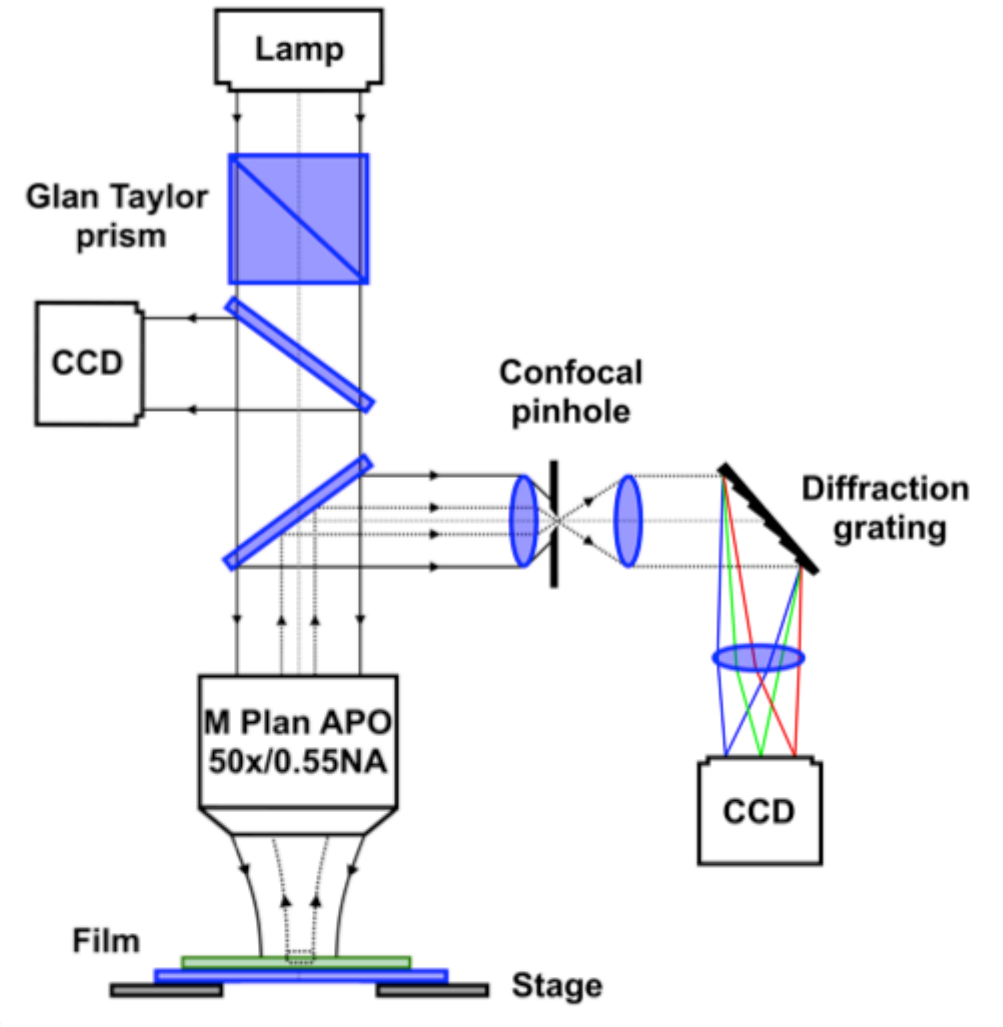

Fig. S2 Reflectance spectroscopy setup for optical characterization of inkjet thin films.

3. X-ray powder diffraction.

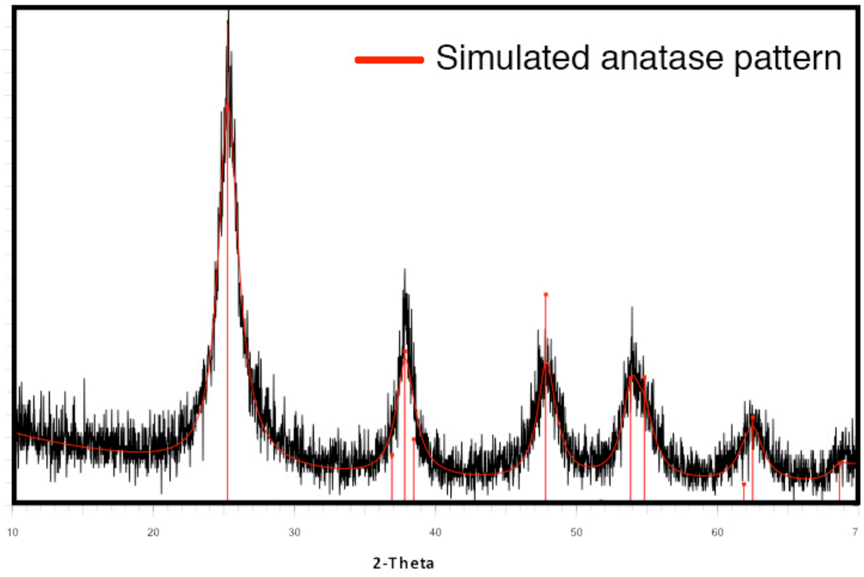

Fig. S3 X-ray diffraction pattern of titania with anatase structure

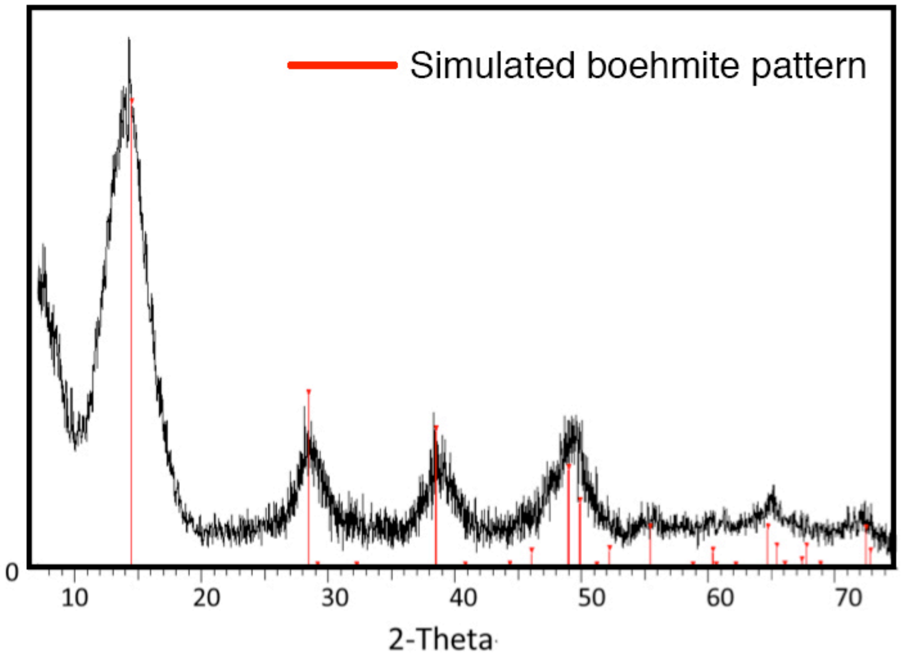

Fig. S4 X-ray diffraction pattern of alumina with boehmite structure
